# Two Novel Antioxidant Nonapeptides from Protein Hydrolysate of Skate (*Raja porosa*) Muscle

**DOI:** 10.3390/md13041993

**Published:** 2015-04-03

**Authors:** Fa-Yuan Hu, Chang-Feng Chi, Bin Wang, Shang-Gui Deng

**Affiliations:** 1 National Engineering Research Center of Marine Facilities Aquaculture, School of Marine Science and Technology, Zhejiang Ocean University, Zhoushan 316022, China; E-Mail: moonriveryue@163.com; 2 School of Food and Pharmacy, Zhejiang Ocean University, Zhoushan 316022, China; E-Mail: wangbin@zjou.edu.cn

**Keywords:** skate (*Raja porosa*), muscle, protein hydrolysate, peptide, antioxidant activity

## Abstract

In the current study, the preparation conditions of neutrase hydrolysate (SMH) from skate (*Raja porosa*) muscle protein were optimized using orthogonal L_9_(3)^4^ tests, and *R* values indicated that pH was the most important factor affecting HO· scavenging activity of SMH. Under the optimum conditions of pH 7.0, enzymolysis temperature 60 °C, enzyme/substrate ratio (E/S) 2%, and enzymolysis time 5 h, EC_50_ of SMH on HO· was 2.14 ± 0.17 mg/mL. Using ultrafiltration, gel filtration chromatography, and RP-HPLC, two novel antioxidant nonapeptides (SP-A and SP-B) were isolated from SMH and their amino acid sequences were found to be APPTAYAQS (SP-A) and NWDMEKIWD (SP-B) with calculated molecular masses of 904.98 Da and 1236.38 Da, respectively. Both showed strong antioxidant activities. SP-A and SP-B exhibited good scavenging activities on HO· (EC_50_ 0.390 and 0.176 mg/mL), DPPH· (EC_50_ 0.614 and 0.289 mg/mL), and O_2_^−^· (EC_50_ 0.215 and 0.132 mg/mL) in a dose-dependent manner. SP-B was also effective against lipid peroxidation in the model system. The aromatic (2Trp), acidic (2Asp and Glu), and basic (Lys) amino acid residues within the sequences of SP-B might account for its pronounced antioxidant activity. The results of this study suggested that protein hydrolysate and peptides from skate muscle might be effective as food additives for retarding lipid peroxidation occurring in foodstuffs.

## 1. Introduction

In food systems, the formation of toxic compounds, such as lipid peroxide and lipid radicals caused by the oxidative deterioration, can decrease food quality and render food unsafe [1,2]. This undesirable process not only produces offensive odors and flavors but also reduces the nutritional content and safety of food by forming secondary reaction products, which reduce shelf life [3]. Furthermore, consuming oxidative foods is thought to cause or increase the risk of heart disease, cancer, stroke, diabetes, and other serious diseases [4]. Inhibiting or slowing down lipid peroxidation by addition of sufficient amounts of antioxidants to preserve the flavor, color, and nutritional value of food would be of considerable value. However, the use of synthetic antioxidants, such as butylated hydroxyanisole, butylated hydroxytoluene (BHT), and tertiary butylhydroquinone, is strictly regulated due to potential health risks [5,6]. Natural antioxidants are in great demand as additives to reduce lipid peroxide and lipid radical activity and improve food quality [7]. The search for safe natural antioxidants has become a research hotspot in the field of biology, medicine, and food science.

Recently, peptides released from various food proteins under controlled endogenous and exogenous enzyme proteolysis have been used as natural antioxidants, generating interest not only in their possibilities as natural alternatives to synthetic antioxidants but also for their beneficial effects, lack of residual side effects, and functionality in food systems [8]. Large quantities of antioxidant peptides have been prepared from proteins from frogs (*Hylarana guentheri*) [9], blue mussels [10], *Sphyrna lewini* [11,12], oysters (*Saccostrea cucullata*) [13], herring [14], and *Meretrix casta* [15]. Preparation of these bioactive hydrolysates and peptides could be affected by different parameters, such as material, protease, and the enzymolysis condition, degree of hydrolysis, peptide structure, and amino acid composition [16,17]. This shows that proper protease hydrolysis processing is important to the preparation of antioxidant peptides.

Cartilaginous fish (*Chondrichthyes*) are a commercially important species. At present, some bioactive components, such as proteins and peptides, glycosaminoglycans, squalenes, and lipids, have been isolated from their muscle, skin, and cartilage [11,18,19,20]. Studies of bioactive protein hydrolysate and peptides from skate (*Raja porosa*) muscle have not been reported until now. In the present study, the preparation conditions of neutrase hydrolysate (SMH) from skate muscle protein were optimized, and two novel antioxidant nonapeptides (SP-A and SP-B) were isolated from SMH and their amino acid sequences were determined, and the antioxidant activities of SP-A and SP-B were evaluated by using assays of DPPH·, HO· and O_2_^−^· scavenging activities and lipid peroxidation inhibition activity.

## 2. Results and Discussion

### 2.1. Preparation of Protein Hydrolysates from Skate Muscle

The types of proteases used for hydrolysis are very important, because different enzymes have different peptide bond cleavage patterns for their substrate, which will have a significant impact on the hydrolytic efficiency, composition, and activities of hydrolysates [21,22]. In the experiment, five familiar proteases were used for the hydrolysis of skate muscle. The enzymolysis conditions, degree of hydrolysis (DH) and HO· scavenging activities of hydrolysates are shown in Table 1. Among the hydrolysates of skate muscle prepared with different enzymes, the neutrase hydrolysate showed the highest DH (14.07% ± 0.86%) and HO· scavenging activities with EC_50_ of 2.57 ± 0.23 mg/mL. These results were consistent with previous reports that the antioxidant capacities of protein hydrolysates were significantly influenced by the enzymes used and positively correlated with the DHs of hydrolysates [16,23]. For these reasons, neutrase was chosen for the preparation of the hydrolysate and its enzymolytic conditions were optimized using an orthogonal L_9_(3)^4^ test.

**Table 1:** Enzymolytic conditions and degree of hydrolysis (DH, %) and HO· scavenging activity (EC_50_ (mg/mL)) Pof protein hydrolysates of skate muscle with five proteinases.

Enzymes	Enzymolysis Conditions			DH (%)	EC_50_ (mg/mL)
	pH	Temp. (°C)	Buffer Solution		
Papain	6.5	50	0.05 M phosphate	12.57 ± 0.65 ^a^	4.66 ± 0.21 ^a^
Flavourzyme	7.0	55	0.05 M phosphate	13.36 ± 0.54 ^b^	3.79 ± 0.16 ^b^
Neutrase	7.0	60	0.05 M phosphate	14.07 ± 0.86 ^c^	2.57 ± 0.23 ^c^
Trypsin	8.0	37	0.05 M Tris-HCl	11.57 ± 0.75 ^d^	5.47 ± 0.31 ^d^
Alcalase	9.5	50	0.05 M Gly-NaOH	13.69 ± 0.93 ^e^	3.06 ± 0.19 ^e^

All the values were mean ± SD (standard deviation). ^a−e^ Values with different letters in the same column indicate significant difference for the same composition determination (*p* < 0.05).

### 2.2. Optimization of Hydrolysis Conditions for Neutrase

The activity of an enzyme is affected by local environmental conditions (such as substrate, liquid/substrate and enzyme/substrate ratios, temperature, pH, and time), which further influence the physicochemical and bioactive properties of the resulting hydrolysates. In the study, the influence of pH, enzyme/substrate ratio (E/S), hydrolysis temperature (Temp.), and time were optimized using HO· scavenging activity as a target. The ranges for orthogonal L_9_(3)^4^ test design were narrowed using single-factor testing (data not shown) [11,12], and the selected variables and their levels are listed in Table 2.

**Table 2:** Range analysis of protein hydrolysates of skate muscle by neutrase on HO· scavenging activities (EC_50_ (mg/mL)) obtained from the L_9_(3)^4^ orthogonal experiment.

No.	Factors				EC_50_ (mg/mL)
	(A) pH	(B) E/S (%)	(C) Time (h)	(D) Temp. (°C)	
1	1 (6.5)	1(1.0)	1 (3)	1 (55)	3.05 ± 0.34 ^a,b^
2	1	2 (1.5)	2 (4)	2 (60)	2.97 ± 0.12 ^b^
3	1	3 (2.0)	3 (5)	3 (65)	2.95 ± 0.29 ^b,c^
4	2 (7.0)	1	2	3	2.86 ± 0.18 ^b,c^
5	2	2	3	1	2.65 ± 0.18 ^d^
6	2	3	1	2	2.39 ± 0.24 ^e^
7	(7.5)	1	3	2	2.78 ± 0.15 ^c,d^
8	3	2	1	3	3.17 ± 0.16 ^a^
9	3	3	2	1	2.87 ± 0.29 ^b,c^
*k*_1_	8.97	8.69	8.61	8.57	
*k*_2_	7.90	8.79	8.70	8.14	
*k*_3_	8.82	8.21	8.38	8.98	
Best level	A_2_	B_3_	C_3_	D_2_	
*K*_1_	2.99	2.90	2.87	2.86	
*K*_2_	2.63	2.93	2.90	2.71	
*K*_3_	2.94	2.74	2.79	2.99	
*R*	0.36	0.19	0.11	0.28	
*R* order	A >D > B >C				

All the values were mean ± SD. *R* referred to the result of extreme analysis. ^a*−*e^ Values with different letters in the same column indicate significant difference for the same composition determination (*p* < 0.05).

The *R* range analysis showed that the influence of four selected factors on HO· scavenging activity (EC_50_) was: A (pH) > D (Temp.) > B (E/S) > C (Time). Hydrolysates (SMH) with the most pronounced antioxidant activity were produced when the hydrolysis conditions for neutrase were A_2_B_3_C_3_D_2_. Under these optimal conditions, the EC_50_ of SMH on HO· scavenging activity was confirmed to be 2.14 ± 0.17 mg/mL, which is to say, the optimum condition of obtaining the highest antioxidative protein hydrolysate of skate muscle was enzymolysis time 5 h, total enzyme dose 2%, enzymolysis temperature 60 °C and pH 7.0.

### 2.3. Purification of Antioxidant Peptides from SMH

#### 2.3.1. Fractionation of SMH by Ultrafiltration

Ultrafiltration is a technique used to separate substances dissolved in solution based on molecular size. It is widely used to purify and concentrate target fraction from protein hydrolysates on the molecular-weight-cutoff (MWCO) membrane. Therefore, the neutrase hydrolysate was fractionated into three different MW fractions through 3 k and 10 kDa ultrafiltration (UF) membranes and named as SMH-I (MW > 10 kDa), SMH-II (MW 3–10 kDa), and SMH-III (MW < 3 kDa), respectively. SMH, SMH-I, SMH-II, and SMH-III scavenged HO· in a concentration-dependent manner with EC_50_ values of 2.14 ± 0.17, 5.93 ± 0.26, 2.47 ± 0.13, and 1.84 ± 0.11 mg/mL, respectively. HO· scavenging assay showed the highest antioxidant capacity for SMH-III with peptides of MW below 3 kDa, followed by SMH-II containing peptides of MW ranged from 3 to 10 kDa. This result was consistent with some earlier reports showing that the antioxidant activity of hydrolysates relied on their MW distributions, and hydrolysates and peptides with lower MW could cross the intestinal barrier more easily [7,16], and interact more effectively with free radicals interfering in oxidative process [24].

#### 2.3.2. Anion-Exchange Chromatography of SMH-III

**Figure 1:**
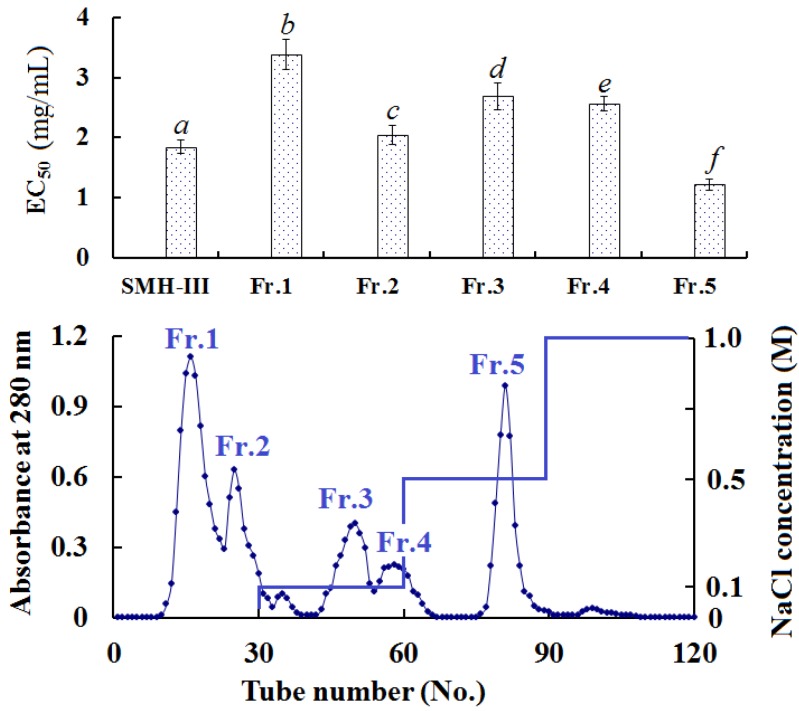
Elution profile of SMH-III as indicated by DEAE-52 cellulose anion-exchange chromatography, and upper panel represented HO· scavenging activity (EC_50_ (mg/mL)). All the values were mean ± SD; SD: standard deviation. (*a*–*f*) Values with same letters indicated no significant difference of different sample at same concentrations (*p* < 0.05).

To produce sub-fractions with more anti-oxidant activity, SMH-III was fractionated using DEAE-52 cellulose anion-exchange chromatography and separated into five fractions (Figure 1). Fr. 1 and Fr. 2 were washed out by deionized water, Fr. 3 and Fr. 4 were washed out by 0.1 M NaCl, and Fr. 5 was washed out by 0.5 M NaCl. Figure 1 indicates that Fr. 5 possessed the highest HO· scavenging rate with EC_50_ of 1.218 ± 0.10 mg/mL among all the samples.

#### 2.3.3. Gel Filtration Chromatography of Fr. 5

**Figure 2:**
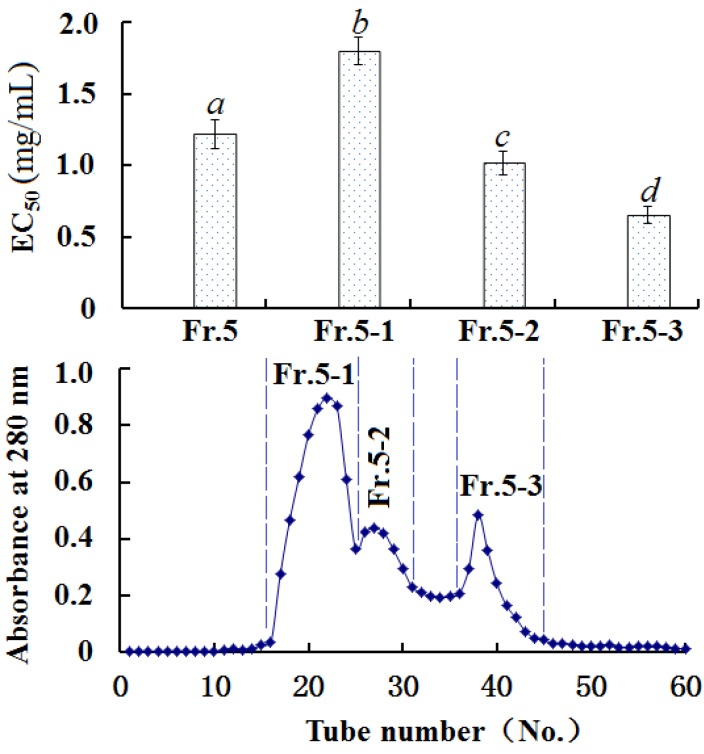
Elution profile of Fr. 5 with size exclusion chromatography on a Sephadex G-15 column, and upper panel represented HO· scavenging activities (EC_50_ (mg/mL)). All the values were mean ± SD. (*a*–*d*) Values with same letters indicated no significant difference of different sample at same concentrations (*p* < 0.05).

As shown in Figure 2, Fr. 5 was separated into three subfractions (Fr. 5-1, Fr. 5-2, and Fr. 5-3). Among the three subfractions, EC_50_ of Fr. 5-3 on HO· was 0.65 ± 0.06 mg/mL, which was lower than those of Fr. 5 (EC_50_ 1.22 ± 0.10 mg/mL), Fr. 5-1 (EC_50_ 1.80 ± 0.10 mg/mL), and Fr. 5-2 (EC_50_ 1.02 ± 0.08 mg/mL), respectively.

#### 2.3.4. Isolation of Peptides from Fr. 5-3 by Reversed-Phase High Performance Liquid Chromatography (RP-HPLC)

**Figure 3:**
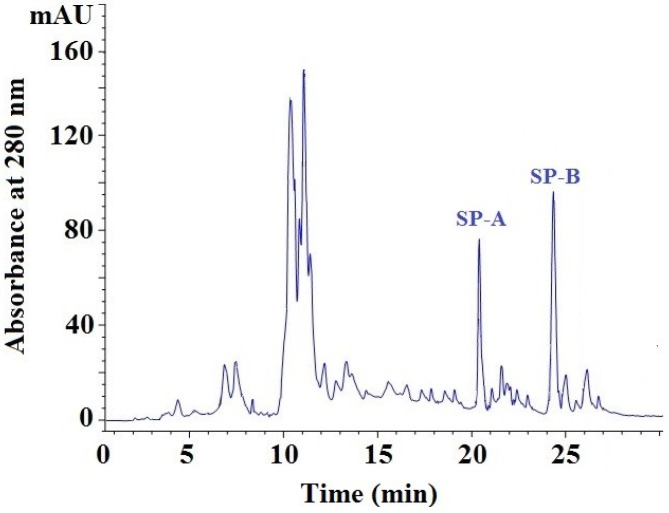
Elution profile of Fr. 5-3 separated by RP-HPLC on a Zorbax, SB C-18 column.

Throughout the purification process, the potent fraction Fr. 5-3 was prepared using a UF membrane system, anion-exchange chromatography and gel filtration chromatography, and it showed high HO· scavenging activity. At last, Fr. 5-3 was further separated by RP-HPLC on a Zorbax, SB C-18 column, and two peptides (SP-A and SP-B) with high purity and content were isolated (Figure 3). Due to lower purity, two larger components, with a retention time of 10–11 min, were not available for subsequent research, and so we will further purify and then identify other antioxidative peptides from the two larger components in future research. EC_50_ values of SP-A and SP-B on HO· were 0.39 ± 0.05 and 0.17 ± 0.02 mg/mL, which was lower than those of SMH-III (1.84 ± 0.11 mg/mL), Fr. 5 (EC_50_ 1.22 ± 0.10 mg/mL), and Fr. 5-3 (EC_50_ 0.65 ± 0.06 mg/mL), respectively.

### 2.4. Amino Acid Sequence Analysis and Molecular Mass Determination

**Figure 4:**
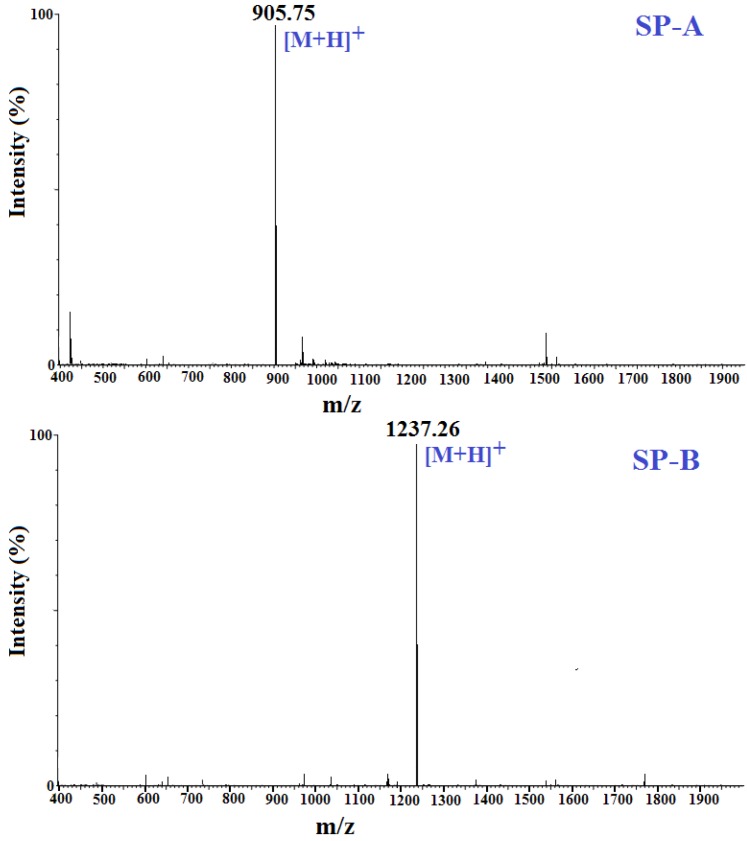
Mass spectra of SP-A and SP-B.

The *N*-terminal amino acid sequences and molecular mass of SP-A and SP-B were determined using a Protein/Peptide Sequencer and Electrospray ionization mass spectrometry (ESI-MS) (Figure 4), respectively. Accordingly, the amino acid sequence of SP-A was determined to be APPTAYAQS, and the detected molecular mass 904.98 Da ([M + H]^+^ 905.75 Da) were closely consistent with the theoretical mass (904.96 Da), calculated from the sequence. The amino acid sequence of SP-B was determined to be NWDMEKIWD and the detected molecular mass 1236.38 Da ([M + H]^+^ 1237.26 Da) agreed well with the theoretical mass (1236.35 Da), calculated from the sequence.

### 2.5. Antioxidant Activity of SP-A and SP-B

**Figure 5:**
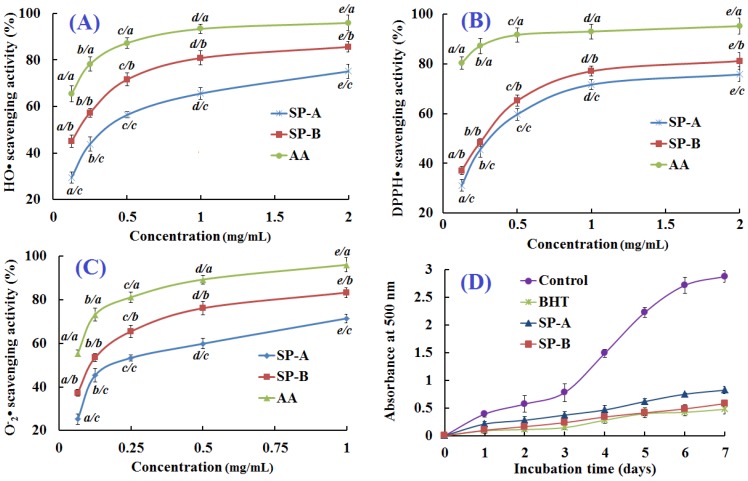
(**A**) HO·; (**B**) DPPH·; and (**C**) O_2_^−^· scavenging activities and (**D**) lipid peroxidation inhibition assays of SP-A and SP-B. All the values were mean ± SD; SD: standard deviation. (*a*–*e*) Values with same letters (the first letter) indicated no significant difference of same sample at different concentrations (*p* < 0.05); (*a*–*c*) Values with same letters (the second letter) indicated no significant difference of different sample at same concentrations (*p* < 0.05).

To evaluate the antioxidant activities of SP-A and SP-B, assays of DPPH·, HO· and O_2_^−^· scavenging activities and lipid peroxidation inhibition activity were performed and the fractions were compared to ascorbic acid (AA) and BHT as positive control (Figure 5).

#### 2.5.1. Hydroxyl Radical Scavenging Activity

HO· is the most reactive of the oxygen radicals, which can react with almost all the substances in the cells and induce severe damage to biomacromolecules in cells, such as enzymes, DNA, and lipids. As shown in Figure 5A, SP-A and SP-B scavenged HO· in a concentration-dependent manner with EC_50_ values of 0.390 and 0.176 mg/mL, respectively. SP-B showed the most pronounced activity of any of the hydrolysates, fractions, or peptides. Many antioxidant peptides have been isolated from other aquatic substances and their by-products. The EC_50_ of SP-B was lower than those of GFRGTIGLVG (0.293 mg/mL) and GPAGPAG (0.240 mg/mL) from protein hydrolysate of croceine croaker scales [2], PSYV (2.64 mg/mL) from protein hydrolysate of loach (*Misgurnus anguillicaudatus*) [25], YPPAK (0.228 mg/mL) from protein hydrolysate of blue mussel (*Mytilus edulis*) [10], PYFNK (0.24 mg/mL) from protein hydrolysate of *Sphyrna lewini* [11], and NGLEGLK (0.313 mg/mL) and NADFGLNGLEGLA (0.612 mg/mL) from protein hydrolysate of giant squid [26,27], but it was higher than those of WDR (0.15 mg/mL) from protein hydrolysate of *S. lewini* [11], GFPSG (0.107 mg/mL) from protein hydrolysate of croceine croaker scales [2], MQIFVKTLTG (0.005 mg/mL) and DLSDGEQGVL (0.007 mg/mL) from protein hydrolysate of venison [28], and NGPLQAGQPGER (0.123 mg/mL) and FDSGPAGVL (0.078 mg/mL) from protein hydrolysate of giant squid [26,27]. SP-B showed good activity against HO·, which indicated that it could be used as the scavenging agent for preventing HO·-induced damage to living cells.

#### 2.5.2. 1,1-Diphenyl-2-picrylhydrazyl (DPPH) Radical Scavenging Activity

Because of its convenience and efficiency, DPPH· scavenging assay is widely used to evaluate the antioxidant ability of protein hydrolysates, fractions, and purified peptides and their suitability for use as free radical scavengers and hydrogen donors. As shown in Figure 5B, SP-A and SP-B were able to reduce stable DPPH· to yellow-colored diphenylpicrylhydrazine. They both showed concentration-dependent anti-DPPH· activity. The EC_50_ of SP-A and SP-B were 0.614 and 0.289 mg/mL, respectively, and SP-B showed most pronounced DPPH· scavenging activity of any of the hydrolysates, fractions, or peptides except for the positive control, AA. The EC_50_ of SP-B was lower than those of GFRGTIGLVG (1.271 mg/mL) and GPAGPAG (0.675 mg/mL) from protein hydrolysate of croceine croaker scales [2], PSYV (17.0 mg/mL) from protein hydrolysate of loach [25], YPPAK (2.62 mg/mL) from protein hydrolysate of blue mussel [10], and WDR (3.63 mg/mL) and PYFNK (4.11 mg/mL) from protein hydrolysate of *S. lewini* [11], but higher than those of GFPSG (0.283 mg/mL) from protein hydrolysate of croceine croaker scales, and VCSV (0.045 mg/mL) and CAAP 0.009 mg/mL) from protein hydrolysate of flounder fish muscle [22]. The data suggested that SP-A and SP-B could convert DPPH· to less harmful or non-harmful products and break the radical chain reaction.

#### 2.5.3. Superoxide Anion Radical Scavenging Activity

Superoxide radicals (O_2_^−^·) can release protein-bound metals and form perhydroxyl radicals, which will initiate lipid oxidation in organism and food systems. The O_2_^−^· scavenging effects of SP-A and SP-B were evaluated at concentrations ranging from 0.065 to 1.000 mg/mL, and the antioxidative activity increased with increasing concentration of the peptides (Figure 5C). SP-A and SP-B could effectively scavenge O_2_^−^· with EC_50_ of 0.215 and 0.132 mg/mL, respectively. The EC_50_ of SP-B was lower than GFRGTIGLVG (0.463 mg/mL) and GFPSG (0.151 mg/mL) from protein hydrolysate of croceine croaker scales [2], and NADFGLNGLEGLA (EC_50_ 0.864 mg/mL) and NGLEGLK (EC_50_ 0.419 mg/mL) from protein hydrolysate of giant squid muscle [27], but it was higher than those of GPAGPAG (0.099 mg/mL) from protein hydrolysate of croceine croaker scales [2], YPPAK (0.072 mg/mL) from protein hydrolysate of blue mussel [10], LKQELEDLLEKQE (EC_50_ 0.128 mg/mL) from protein hydrolysate of oyster [29], and WDR (EC_50_ 0.09 mg/mL) and PYFNK (EC_50_ 0.11 mg/mL) from protein hydrolysate of *S. lewini* [11]. In living cells, the neutralization of O_2_^−^· to hydrogen peroxide can be catalyzed by superoxide dismutase (SOD), which is one of the defense mechanisms of cytoprotection against reactive oxygen. SP-B showed strong O_2_^−^· scavenging activity and might have SOD-like activity, thereby scavenging O_2_^−^· in biological organisms.

#### 2.5.4. Lipid Peroxidation Inhibition Assay

Each radical scavenging assay represented only one kind of antioxidant mechanism, which could not reflect the comprehensive capacity of samples that acted as antioxidants to retard or inhibit lipid oxidation in biological organisms or food systems. Lipid peroxidation is a complicated process involving the formation and propagation of lipid radicals and hydroperoxides in the presence of oxygen. The activities of SP-A and SP-B against the peroxidation of linoleic acid were investigated and compared to that of BHT in a subsequent experiment.

As shown in Figure 5D, the control (not exposed to antioxidant) had the highest absorbance value in the test after a seven-day incubation, indicating the highest degree of oxidation among the samples, but SP-A and SP-B inhibited lipid peroxidation in linoleic acid emulsion system effectively for up to seven days. The activity of SP-B was similar to that of positive control (BHT). Results indicated that SP-B had the same comprehensive capacity as BHT in the linoleic acid peroxidation system.

### 2.6. Relationship between Antioxidant Activities and Amino Acid Compositions of Peptides

The antioxidant activities of peptides may rely on molecular size, amino acid sequence, and the presence of amino acids capable of involvement in oxidative reactions [30,31]. In context, the amino acid compositions of SP-A (APPTAYAQS) and SP-B (NWDMEKIWD), such as hydrophobic/aromatic, acidic, and basic amino acids, were different. This could be the main cause of their antioxidant properties.

Antioxidant peptides possess some metal chelation and hydrogen- and electron-donating activity, which allows them to interact with free radicals and either of them terminates radical chain reactions or prevents them from taking place [25,32]. Sarmadi and Ismail reported that aromatic amino acids such as Trp, Tyr, and Phe, could donate protons to electron-deficient radicals and therefore enhanced the radical scavenging activities of peptides [33]. Dávalos *et al.* reported that Met, Trp, and Tyr showed the most pronounced antioxidant activity of any amino acids [34]. Ren *et al.* reported that one or more residues of Trp, Met, Tyr, His, Pro, Cys, and Phe could enhance the activity of antioxidant peptides [32]. This finding was further supported by the research reported by Zhang, Mu, and Sun, who found that antioxidant amino acids, such as Trp, Met, Tyr, and Phe, were responsible for their activities of the identified peptides (YYIVS, TYQTF, SGQYFL, YYDPL and YMVSAIWG) [35]. One Tyr and two Pro in sequences of SP-A (APPTAYAQS) and two Trp and one Met in sequence of SP-B (NWDMEKIWD) should be responsible for their high antioxidant activities.

In many previous studies, hydrophobic amino acids were considered the main factors affecting the antioxidant activities of peptides [25,36,37]. Although, there were more hydrophobic amino acids in the sequence of SP-A (2Pro, 3Ala, and Tyr) than those in the SP-B (2Trp, Ile, and Met), SP-A showed less free radical scavenging and less lipid peroxidation inhibition activity than SP-B in the current study. There might be other factors affecting the activity of SP-B. Carefully analyzing the amino acid compositions of SP-A and SP-B, the significant difference between the two peptides was the presence of basic (Lys) and acidic (2Asp and Glu) amino acid residues in the sequence of SP-B. Byun *et al.* and Gimenez *et al.* have reported that acidic and basic amino acid residues played a role in metal ion chelating activity, which is related to antioxidant activity [38,39]. Ren *et al.* have reported that basic peptides had greater capacity to scavenge hydroxyl radicals than acidic or neutral peptides [32]. In addition, Klompong *et al.* reported that the ratio of hydrophilic and hydrophobic forces of peptides had another crucial influence on the peptide solubility in aqueous solution and lipids [23]. It might be speculated that the acidic and basic amino acid residues in SP-B made it easier for the structure to interact with free radicals and exert antioxidant activity at the cost of their own antioxidant activity. SP-B was found to have more long side chains than SP-A did. Carboxyl and amino groups in the side chains can act as hydrogen donors and metal ion chelators [40]. These findings were expected because amino acids with long side chains can interact with free radicals very effectively and inhibit the propagation cycles of lipid peroxidation.

## 3. Experimental Section

### 3.1. Materials

The frozen skate (*R. porosa*) was purchased from Nanzhen market in Zhoushan City, Zhejiang Province, China, and authenticated by Sheng-long Zhao (Zhejiang Ocean University, Zhoushan, China). Trypsin, flavourzyme, neutrase, papain, alcalase, 2,2-diphenyl-1-picrylhydrazyl (DPPH), Sephadex G-15, and trinitrobenzene sulfonic acid (TNBS) were purchased from Sigma-Aldrich (Shanghai, China) Trading Co., Ltd. Acetonitrile was LC grade and purchased from Thermo Fisher Scientific Co., Ltd. (Shanghai, China). All other reagents used in the experiment were of analytical grade and purchased from Sinopharm Chemical Reagent Co., Ltd. (Shanghai, China).

### 3.2. Protein Hydrolysates of Skate Muscle (SMHs) Using Proteases

#### 3.2.1. Preparation of Protein Hydrolysates Using Five Proteases

The skate muscle was separated manually, pounded to homogenate, and defatted as described in a previous work [10]. The homogenate was mixed with isopropanol at a ratio of 1:4 (*w/v*), homogenized, and allowed to stand at 35 °C for 1 h. The mixture was filtered and the resulted residue was defatted at 35 °C for 1.5 h using isopropanol at a ratio of 1:4. Finally, the mixture was centrifuged at 4500× *g* for 15 min and the precipitate (defatted protein) was freeze-dried and stored at −20 °C.

Defatted protein was dissolved in different buffer solution at a ratio of 1:4 (*w/v*) and hydrolyzed for 3 h separately using five proteases with an enzyme/substrate (E/S) ratio of 1.5% (*w/w*) (Table 1). Hydrolysate samples were taken out, inactivated in boiling water bath for 10 min, and centrifuged at 10,000× *g* for 15 min. The resulting supernatants were prepared for measurement of the degree of hydrolysis (DH) and HO· scavenging activity.

#### 3.2.2. Optimum Conditions for the Active Enzymatic Hydrolysate

To prepare defatted protein hydrolysates of skate muscle; enzymatic hydrolysis was performed using neutrase. An orthogonal L_9_(3)^4^ test design was used to optimize the conditions of enzymatic hydrolysis [11,12]. Four controllable variables, including pH, enzyme/substrate ratio (E/S; %), time (h), and temperature (°C), were selected. The selected variables and their levels were listed in Table 2. Defatted protein (5 g) was put into 20 mL distilled water and hydrolyzed with neutrase according to the experimental runs (Table 2). The subsequent processing was the same as the method described at the section of preparation of protein hydrolysates using five proteases. The hydrolysate prepared under the optimum conditions was referred to as SMH.

### 3.3. Isolation of Antioxidant Peptides from SMH

#### 3.3.1. Fractionation of SMH by Ultrafiltration

SMH solution was filtered using two ultrafiltration membranes (Diaflo membranes, 76 mm diameter, MilliporeGlostrup, Denmark) with 3 and 10 kDa MWCO to produce three fractions corresponding to molecular weights (MW) above 10 kDa (SMH-I), between 3 and 10 kDa (SMH-II), and below 3 kDa (SMH-III). Three fractions were dialyzed, concentrated, and lyophilized. SMH-III was chosen for further study on HO· scavenging activity.

#### 3.3.2. Anion-Exchange Chromatography

Anion-exchange chromatography was performed according to the procedure of Wang *et al.* [11]. SMH-III solution (5 mL, 30.0 mg/mL) was added into a pre-equilibrated DEAE-52 cellulose (Shanghai Yuanju Biological Technology Co., Ltd., Shanghai, China) column (1.6 × 60 cm) with distilled water, and stepwise eluted with 150 mL deionized water to 0.1, 0.5, and 1.0 M NaCl solutions, respectively, with a flow rate of 1.0 mL/min. Each eluted fraction (5 mL) was collected and monitored at 280 nm, and five fractions (Fr. 1 to Fr. 5) were pooled, desalted using D101 macroporous resin (Tianjin Bohong Resin Technology Co., Ltd., Tianjin, China), lyophilized, and measured their HO· scavenging activities.

#### 3.3.3. Gel Filtration Chromatography of SMH-III

Gel filtration chromatography was performed according to the procedure of Luo *et al.* [12]. Fr. 5 (100.0 mg) was dissolved in 5 mL deionized water and purified by Sephadex G-15 gel filtration column (2.6 × 80 cm) equilibrated previously with deionized water. The column was eluted with deionized water at a flow rate of 1 mL/min, and the eluate was collected every 3 mL and measured at 280 nm. Three subfractions (Fr. 5-1, Fr. 5-2, and Fr. 5-3) were collected, lyophilized, and their HO· scavenging activities were determined.

#### 3.3.4. Isolation of Peptides from Fr. 5-3 by RP-HPLC

The subfraction of Fr. 5-3 was further separated by reverse-phase high-performance liquid chromatography (RP-HPLC) (Agilent 1200 HPLC, Agilent Ltd., Santa Clara, CA, USA) according to the procedure of Wang *et al.* [11]. Fr. 5-3 (10.0 mg) was dissolved in 1 mL of 0.1% trifluoroacetic acid (TFA), and the resulting solution (20 μL) was injected into a Zorbax, SB C-18 column (column size: 4.6 mm × 250 mm, 5 μm particle size, Agilent), using 30% acetonitrile containing 0.1% TFA at a flow rate of 1.0 mL/min. The eluate was detected at 280 nm and two peptides, here called SP-A and SP-B, were isolated, collected, and lyophilized.

### 3.4. Degree of Hydrolysis (DH)

DH was determined according to the method described by Chi *et al.* [21]. Hydrolysate (50 μL) was mixed with 0.5 mL of phosphate buffer (0.2 M, pH 8.2) and 0.5 mL of TNBS reagent (0.05%) freshly prepared by diluting with deionized water. After being incubated at 50 °C for 1 h in a water bath, the reaction in mixture was stopped by adding 1 mL of HCl (0.1 M) and incubated at 25 °C for 30 min. The absorbance was measured at 420 nm. l-Leucine was used as a standard. To determine the total amino acid content, defatted skate muscle was completely hydrolyzed with 6 M HCl with a sample/acid ratio of 1:100 at 120 °C for 24 h. DH (%) was calculated using the following equation:

DH = [(A_t_ − A_0_)/(A_max_ − A_0_)] × 100% (1)

Where, A_t_ is the number of α-amino acids released at time t, A_0_ is the number of α-amino acids in the supernatant at 0 h, and A_max_ is the total number of α-amino acids obtained after acid hydrolysis at 120 °C for 24 h.

### 3.5. Amino Acid Sequence Analysis and Molecular Mass Determination

The antioxidant peptides of SP-A and SP-B were subjected to *N*-terminal amino acid sequencing on an Applied Biosystems 494 protein sequencer (Perkin Elmer Wallace Inc., Akron, OH, USA) according to the previous method [2]. Edman degradation was performed according to the standard program supplied by Applied Biosystems.

Accurate molecular masses of SP-A and SP-B were determined using a Q-TOF mass spectrometer (Micromass, Waters, Los Angeles, CA, USA) coupled with an electrospray ionization (ESI) source on the previous method developed by Luo *et al.* [11]. Ionization was carried out in positive mode with a capillary voltage of 3500 V. Nitrogen was maintained at 40 psi for nebulization and 9 L/min at 350 °C for evaporation temperature. Data were collected in centroid mode from *m/z* 100 to 2000.

### 3.6. Antioxidative Activity

The DPPH·, HO· and O_2_^−^· scavenging activities were measured according to the method described by Wang *et al.* [11], and the results were expressed as a half elimination ratio (EC_50_) defined as the concentration where a sample caused a 50% decrease of the initial concentration of DPPH·, HO· and O_2_^−^·.

#### 3.6.1. HO· Scavenging Activity

HO· scavenging activity was measured using the method developed by Wang *et al.* [10]. In this system, HO· is generated by the Fenton reaction. HO· can oxidize Fe^2+^ to Fe^3+^, and only Fe^2+^ can combine with 1,10-phenanthroline to form a red compound (1,10-phenanthroline-Fe^2+^). It shows maximum absorbance at 536 nm. The concentration of HO· is reflected by the degree of decolorization of the reaction solution. Briefly, 1,10-phenanthroline solution (1.0 mL, 1.865 mM) and the sample (2.0 mL) were added into a screw-capped tube and mixed. The FeSO_4_·7H_2_O solution (1.0 mL, 1.865 mM) was then pipetted into the mixture. The reaction was initiated by adding 1.0 mL H_2_O_2_ (0.03% *v/v*). After incubation at 37 °C for 60 min in a water bath, the absorbance of the reaction mixture was measured at 536 nm against a reagent blank. Reaction mixture without any antioxidant served as the negative control, and mixture without H_2_O_2_ served as the blank. HO· scavenging activity (HRSA) was calculated by the following formula:

HRSA(%) = [(A_s_ − A_c_)/(A_b_ − A_c_)] × 100% (2)

where A_s_ is the sample absorbance; A_c_ is the control group absorbance; and A_b_ is the blank absorbance.

#### 3.6.2. DPPH· Scavenging Activity

DPPH· scavenging activity was tested as previously described [10]. Two milliliters of deionized water containing different concentrations of sample (from 0.125 to 2.0 mg/mL) were placed in the cuvettes, and then 500 μL of ethanol solution of DPPH (0.02%) and 1.0 mL of ethanol were added. The control containing DPPH solution without sample was also prepared. In blank, DPPH solution was replaced with ethanol. The antioxidant activity of the sample was evaluated using the relative amount of inhibition of DPPH· with the following equation:

DPPH· scavenging activity (%) = (A_c_ + A_b_ − As)/A_c_ × 100% (3)

where A_s_ is the sample absorbance; A_c_ is the control group absorbance; and A_b_ is the blank absorbance.

#### 3.6.3. O_2_^−^· Scavenging Activity

The O_2_^−^· scavenging activity of the sample was investigated by the previous method [21]. In the experiment, O_2_^−^· was generated in 1 mL of nitrotetrazolium blue chloride (2.52 mM), 1 mL of NADH (624 mM), and 1 mL of different concentrations of samples (from 0.065 to 1.0 mg/mL). The reaction was initiated by adding 1 mL of phenazine methosulfate solution (120 μg/mL) to the reaction mixture. The absorbance was measured at 560 nm against the corresponding blank after 5 min incubation at 25 °C. The capacity of scavenging O_2_^−^· was calculated using the following equation:

O_2_^−^· scavenging activity (%) = [(A_c_ − A_s_)/A_c_] × 100% (4)

where A_s_ is the absorbance with sample; and A_c_ is the absorbance without sample.

#### 3.6.4. Lipid Peroxidation Inhibition Assay

The assay was performed according to the method described by Wang *et al.* [10]. A sample (5.0 mg) was dissolved in 10 mL of phosphate buffer (0.05 M, pH 7.0), and added to a solution of 0.13 mL of linoleic acid and 10 mL of 99.5% ethanol. Then, the total volume was adjusted to 25 mL with deionized water. The mixture was incubated in a conical flask with a screw cap at 40 ± 1 °C in a dark room, and the degree of linoleic acid oxidation was evaluated at 24-h intervals by measuring the ferric thiocyanate values according to the method of Mitsuta *et al.* [41]. The reaction solution (100 μL) incubated in the linoleic acid model system was mixed with 4.7 mL of 75% ethanol, 0.1 mL of 30% ammonium thiocyanate, and 0.1 mL of 20 mM ferrous chloride solution in 3.5% HCl. After 3 min, the thiocyanate value was determined by reading the absorbance at 500 nm following color development with FeCl_2_ and thiocyanate at different intervals during the incubation period at 40 ± 1 °C.

### 3.7. Statistical Analysis

All experiments were performed in triplicate (*n* = 3) and all the values were mean ± SD (standard deviation). ANOVA test (using SPSS 13.0 software) was used to determine the mean value of each treatment. Significant differences between the means of parameters were determined using Duncan’s multiple range test (*p* < 0.05).

## 4. Conclusions

The present study is the first to report optimized conditions for the preparation of neutrase hydrolysate (SMH) from skate (*R. porosa*) muscle protein using orthogonal L_9_(3)^4^ tests. pH value was found to be the most important factor affecting HO· scavenging activity of SMH based on the *R* values. Using ultrafiltration and connective chromatographic methods, two novel nonapeptides with strong antioxidant activity, SP-A and SP-B, were isolated from SMH, and their amino acid sequences were found to be as APPTAYAQS (SP-A) and NWDMEKIWD (SP-B). The current results indicated that SMH and the two purified peptides might be beneficial as food additives and diet nutrients and serve preventative roles in oxidative reactions. Further research should be performed to illustrate their mechanism of action and functionality-structure relationship.
